# Genome and Karyotype Reorganization after Whole Genome Duplication in Free-Living Flatworms of the Genus *Macrostomum*

**DOI:** 10.3390/ijms21020680

**Published:** 2020-01-20

**Authors:** Kira S. Zadesenets, Ilyas Y. Jetybayev, Lukas Schärer, Nikolay B. Rubtsov

**Affiliations:** 1 The Federal Research Center Institute of Cytology and Genetics SB RAS, Lavrentiev ave. 10, 630090 Novosibirsk, Russia; jetybayev@bionet.nsc.ru; 2 Evolutionary Biology, Zoological Institute, University of Basel, CH-4051 Basel, Switzerland; lukas.scharer@unibas.ch; 3 Department of Cytology and Genetics, Novosibirsk State University, Pirogova str. 2, 630090 Novosibirsk, Russia

**Keywords:** karyotype instability, karyotypic and genomic diversity, genome evolution, repetitive DNA, aneuploidy, whole genome duplication, polyploidy, flatworms

## Abstract

The genus *Macrostomum* represents a diverse group of rhabditophoran flatworms with >200 species occurring around the world. Earlier we uncovered karyotype instability linked to hidden polyploidy in both *M. lignano* (2*n* = 8) and its sibling species *M. janickei* (2*n* = 10), prompting interest in the karyotype organization of close relatives. In this study, we investigated chromosome organization in two recently described and closely related *Macrostomum* species, *M. mirumnovem* and *M. cliftonensis*, and explored karyotype instability in laboratory lines and cultures of *M. lignano* (DV1/10, 2*n* = 10) and *M. janickei* in more detail. We revealed that three of the four studied species are characterized by karyotype instability, while *M. cliftonensis* showed a stable 2*n* = 6 karyotype. Next, we performed comparative cytogenetics of these species using fluorescent in situ hybridization (FISH) with a set of DNA probes (including microdissected DNA probes generated from *M. lignano* chromosomes, rDNA, and telomeric DNA). To explore the chromosome organization of the unusual 2*n* = 9 karyotype discovered in *M. mirumnovem*, we then generated chromosome-specific DNA probes for all chromosomes of this species. Similar to *M. lignano* and *M. janickei*, our findings suggest that *M. mirumnovem* arose via whole genome duplication (WGD) followed by considerable chromosome reshuffling. We discuss possible evolutionary scenarios for the emergence and reorganization of the karyotypes of these *Macrostomum* species and consider their suitability as promising animal models for studying the mechanisms and regularities of karyotype and genome evolution after a recent WGD.

## 1. Introduction

Modern ideas about the mechanisms of evolution of eukaryotes are based mostly on Darwin’s theory of natural selection, Kimura’s neutral theory (as well as Ohta’s “nearly neutral” theory), and a set of gene transmission rules [1,2]. Specifically, the gradual accumulation of mutations (deleterious, neutral, or advantageous) followed by natural selection and genetic drift is considered the main mechanism underlying the gradual evolution of most life forms. Only some of the mutations are thought to be advantageous and increase an organism’s fitness and, therefore, can be positively selected. Nevertheless, mutations are considered the main generator of essential genetic variation for further natural selection or neutral drift, eventually leading to new life forms and speciation, mostly in the long term [3].

More recently, alternative mechanisms that lead to genetic innovation and variation have come into focus, which involve different forms of genome reorganization, including whole genome duplication (WGD) followed by genome rediploidization [4,5]. A WGD could protect an organism from the effects of some types of deleterious mutations, by doubling all genes of the ancestral genome through either auto- or allotetraploidization [6,7]. Autotetraploids arise due to genome doubling within the same species, i.e., the newly formed genome consists of two copies of the original diploid genome. Traces of ancient WGDs were detected in different taxa of eukaryotes, including ciliates (*Paramecium tetraurelia*), fungi (*Saccharomyces cerevisiae*), animals, and, most prominently, plants [8,9,10,11]. In some phylogenetic lineages of plants, modern genomes were formed as a result of several (up to six) rounds of WGD events [12]. In invertebrates, WGD was discovered in, for example, bdelloid rotifers [13] and chelicerates (horseshoe crabs and the common house spider, *Parasteatoda tepidariorum*) [12,14]. At the evolutionary origin of vertebrates, there were two WGD events, with additional events occurring among some groups of teleost fishes (e.g., the family Salmonidae experienced two more rounds of WGD) [15]. Most of these WGD events were probably associated with autopolyploidy, but in the genome evolution of some representatives of fishes (for instance, the goldfish *Carassius auratus*), amphibians (African clawed frog *Xenopus laevis*), and reptiles (*Ambystoma* salamanders), WGDs took place after interspecific hybridization [16,17,18,19]. In contrast to autopolyploids, such allotetraploids are formed through WGD of the hybrid genome, and they consequently contain both parental subgenomes [6]. WGD after interspecific hybridization is widespread in different groups of organisms, including yeasts, plants, insects, amphibians, reptiles, and fishes [20,21]. Furthermore, in some taxa, its frequency appears to be rather high [20,22]. The main mechanism for the duplication of hybrid genomes is considered to be the fusion of unreduced gametes [21]. The genomes and karyotypes of such allopolyploids were observed to be less stable compared to autopolyploids and were usually found to be significantly reorganized [16,17,23].

A WGD provides large amounts of genetic raw material, which can be involved in subsequent evolutionary adaptation and innovation [15]. It is known that the vast majority of gene duplicates usually get lost within a few million years, while extant genes in duplicate copies (ohnologs) are usually under strong purifying selection [24]. This leads to significant and dynamic reorganization of the doubled genome, which may eventually reach a re-diploidized state [17,25,26]. Although the long-term processes after WGD, including maintenance, inactivation, and diversification in the function of gene duplicates are comparatively well studied [27], the processes that take place immediately after a WGD are not well studied and understood. There are relatively few known species that have undergone a recent WGD in their evolution, thus permitting studies of the short-term evolutionary processes that occur immediately after a WGD.

Among animals, these studies are mostly restricted to allopolyploid genomes of amphibians (e.g., *Xenopus laevis* and the *Pelophylax esculentus* complex) [17,28] and bony fishes (e.g., *Carassius auratus*, *Cyprinus carpio*) [18,26,29]. These hybrid genomes contain an expanded pool of genes derived from the genomes of their parental species, and it has been argued that they may therefore show increased potential for evolutionary change [26,30], due to the presence of homeologous genes and a high level of genomic instability induced by a burst of transposable element (TE) activity following hybridization. TE activity and the subsequent expansion of repetitive DNA sequences could lead to rapid genome remodeling, including massive gene losses through numerous chromosomal rearrangements (including deletions, inversions, and translocations of chromosomal regions) and ectopic recombination [31,32]. This suggests that species that have undergone a recent WGD could be useful model organisms in studies of macroevolutionary processes. Unfortunately, amphibians are characterized by very large genomes, while karyotypes of bony fishes are characterized by very high chromosome numbers [33]. The available species with smaller genome sizes and lower chromosome numbers (i.e., species having characteristics that would facilitate genomic and karyotype analyses) are mostly paleoploids, which already carry significantly reorganized genomes, presenting numerous problems in determining which parental species a gene originated from, and by what route the genome changes occurred.

Several closely related species belonging to the free-living flatworm genus *Macrostomum*, the subject of our study here, are a fortuitous exception to this pattern [34] (see also Figure S1). All *Macrostomum* species analyzed to date are characterized by relatively low chromosome numbers, ranging from generally 2*n* = 6 to more rarely 2*n* = 12 [35,36], and they have fairly small genome sizes of <1000 Mbp, going as low as 217 Mbp [34,37]. Crucially, at least three *Macrostomum* species have undergone recent WGD events. Previously, we showed that *Macrostomum lignano* and *M. janickei* (then called *Macrostomum* sp. 8) underwent a recent WGD event, probably through autopolyploidization [38,39]. As we show here, the recently described *M. mirumnovem* [34] appears to represent another case of a recent WGD, followed by considerable genome destabilization. To document this in more detail, we carried out karyological and comparative molecular cytogenetic analyses of these three species and compared the patterns to those of a fourth closely related species, *M. cliftonensis* [34], showing a small genome with a canonical 2*n* = 6 chromosome number, which suggests that its genome is in a pre-WGD state.

## 2. Results

### 2.1. Single-Worm Karyotyping of Four Macrostomum Species

#### 2.1.1. *Macrostomum lignano*

**Figure 1:**
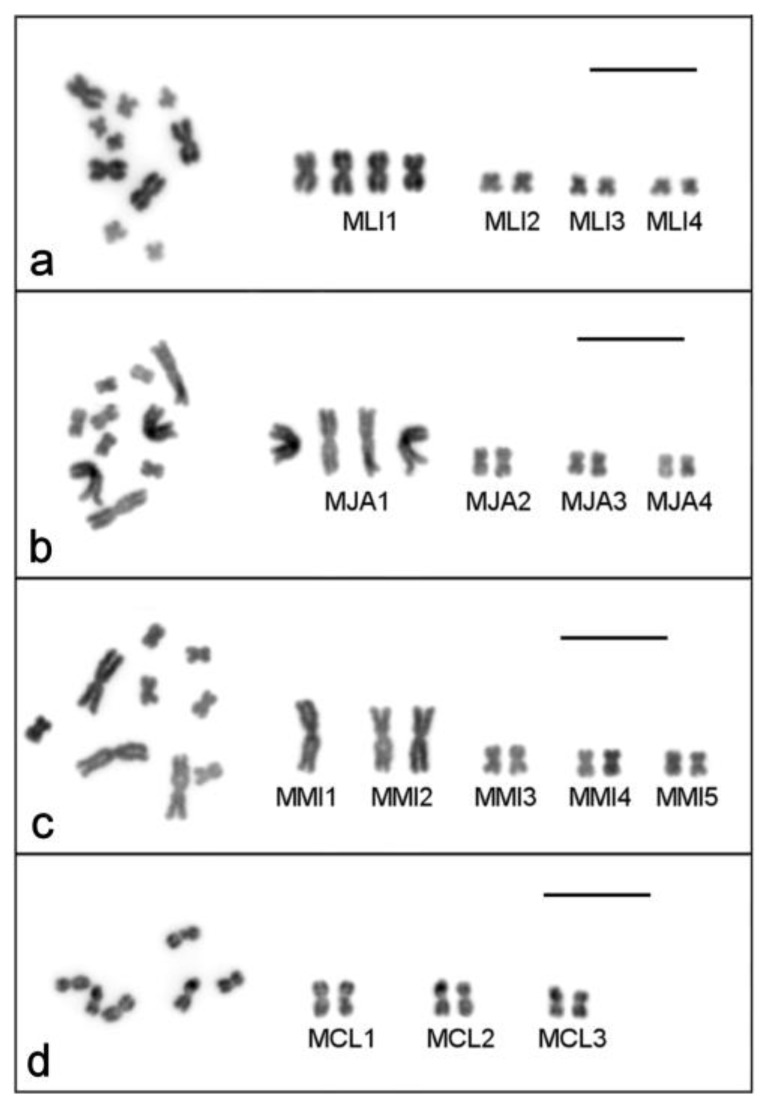
Metaphase plates (left) and karyotypes (right) of the four studied *Macrostomum* species: (**a**) 2*n* = 10 karyotype of inbred DV1/10 line of *M. lignano*; (**b**) 2*n* = 10 karyotype of *M. janickei*; (**c**) 2*n* = 9 karyotype of *M. mirumnovem*; (**d**) 2*n* = 6 karyotype of *M. cliftonensis*. 4′,6-diamidino-2-phenylindole (DAPI)-staining (inverted image). Scale bar 10 µm.

**Figure 2:**
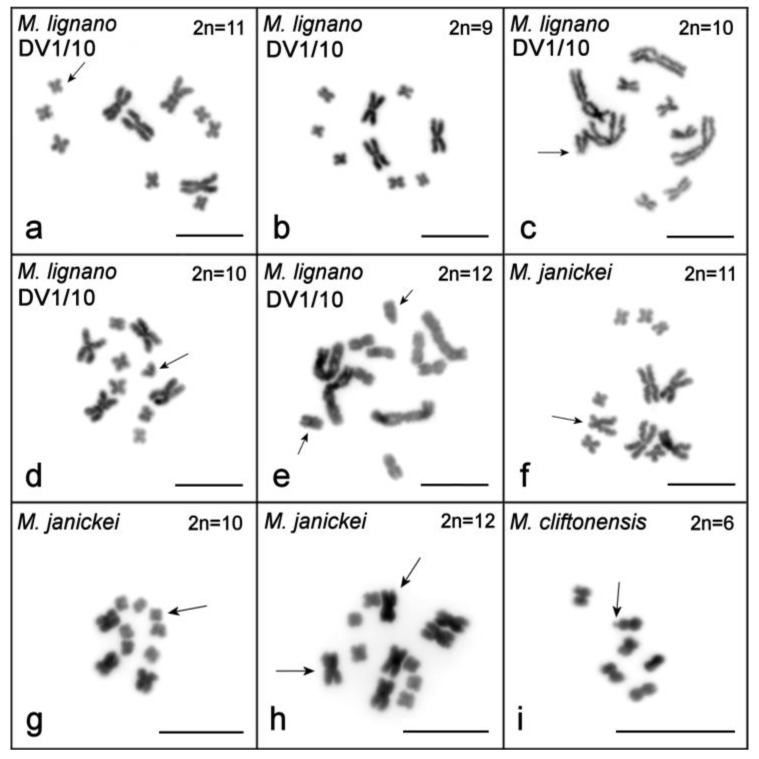
Metaphase plates from specimens with abnormal karyotypes: (**a**–**e**) DV1/10 line of *M. lignano*, (**f**–**h**) *M. janickei*, and (**i**) *M. cliftonensis*. (**a**) Additional small metacentric chromosome; (**b**) one copy of the large chromosome is lost; (**c**) additional small submetacentric chromosome (small extra chromosome or rearranged homolog of small chromosome MMI4); (**d**) additional small acrocentric chromosome (small extra chromosome or rearranged homolog of small chromosome MMI4); (**e**) two small extra chromosomes (one metacentric and one submetacentric); (**f**) one medium-sized submetacentric chromosome; (**g**) one small extra chromosome; (**h**) two additional large metacentric chromosomes; (**i**) metaphase plate of *M. cliftonensis* with chromosome showing secondary constriction in one homolog of MCL1. Chromosomal abnormalities and secondary constriction are marked with arrows. Scale bar 10 µm.

The karyotype of *M. lignano* was previously described as 2*n* = 8, with one pair of large and three pairs of small metacentric chromosomes [35]. However, many laboratory lines and cultures, as well as some specimens freshly collected from the field, were subsequently shown to harbor polymorphisms for the number of chromosomes between individual worms (with no mosaicism observed in ≥10 analyzed metaphase plates per individual), with the main variation concerning the number of large chromosomes (Table 1) [36]. For the analysis of karyotype stability in the newly established DV1/10 inbred line, we karyotyped ~100 individual worms every year. Results of the screening suggested that this line was karyologically quite stable (Table 1). The last karyotyping of 100 worms in spring 2018 revealed that 95 individuals showed the 2*n* = 10 karyotype (Figure 1a), while structural and numerical chromosome aberrations were observed in five specimens (Figure 2a–e). Therefore, the karyotype stability in this line appeared to be greater than that in the original inbred DV1 line [38,39], which was also karyotyped multiple times (Table 1).

#### 2.1.2. *Macrostomum janickei*

To date, the most frequently observed karyotype in *M. janickei* (2*n* = 10) contained four large and six small metacentric chromosomes (Table 1) [36], and this karyotype was identical to the karyotype of the DV1/10 line with respect to chromosome morphology and painting with microdissected DNA probes [38,39]. However, karyotyping of the laboratory culture of *M. janickei* performed in 2018 showed that among 100 analyzed specimens only 48 had the ordinary 2*n* = 10 karyotype (Figure 1b), while 39 showed the 2*n* = 11 karyotype with one additional large chromosome. The karyotype of two worms included only three copies of large chromosomes instead of the usual four (2*n* = 9). The remaining 11 specimens were characterized by various numerical and structural chromosomal abnormalities (Table 1). Some of them are shown in Figure 2f–h.

#### 2.1.3. *Macrostomum mirumnovem*

**Figure 3:**
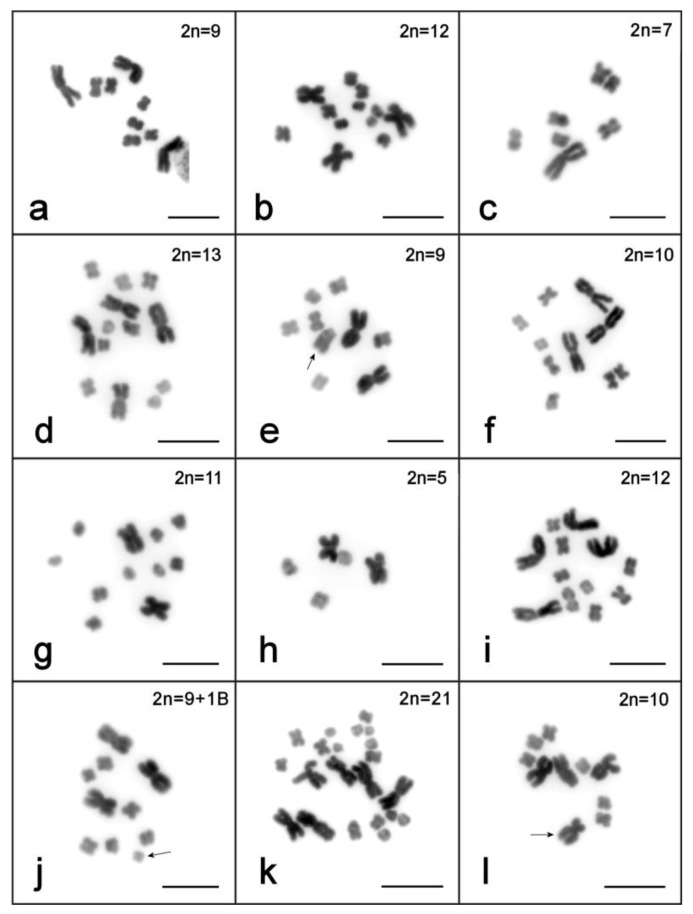
Karyotype variation in *M. mirumnovem* specimens: (**a**) karyotype 9,–MMI1; (**b**–**l**) karyotypes different from 9,–MMI1. (**b**) 2*n* = 12 (three large and nine small metacentrics); (**c**) 2*n* = 7 (one large and six small metacentrics); (**d**) 2*n* = 13 (four large and nine small metacentrics); (**e**) 2*n* = 9 (two large and six small metacentrics and one medium-sized submetacentric, marked with an arrow); (**f**) 2*n* = 10 (three large and seven small metacentrics); (**g**) 2*n* = 11 (two large and nine small metacentrics); (**h**) 2*n* = 5 (two large and three small metacentrics); (**i**) 2*n* = 12 (four large and eight small metacentrics); (**j**) 2*n* = 9 + 1B (three large and six small metacentrics and one B chromosome, marked with an arrow); (**k**) 2*n* = 21 (six large and 15 small chromosomes); (**l**) 2*n* = 10 (three large metacentrics and six small metacentrics, and one medium-sized submetacentric, marked with an arrow).

The initial karyotyping of 52 specimens was performed after three months of cultivation under standard laboratory conditions (~4 generations). Highly variable chromosome sets in karyotyped specimens were revealed (Table 1), and all observed karyotypes were asymmetric (i.e., contained chromosomes with highly heterogeneous sizes). Like the karyotypes of *M. lignano* and *M. janickei*, they contained both small and large chromosomes, with no intermediate-sized ones. The most frequent karyotype, observed in 34 specimens (65.4%), consisted of one unpaired largest metacentric chromosome (chromosome #1, MMI1), a pair of large metacentrics (chromosome #2, MMI2), and three pairs of small metacentrics (MMI3–MMI5) (Figure 1c). Due to the observed variability of the *M. mirumnovem* karyotype, describing the normal karyotype presents a problem. Thus, to describe the obtained karyotypes we use the hypothetical modal complete karyotype (HMCK) concept, which for *M. mirumnovem* contains five chromosome pairs including two copies of MMI1, and indicate deviations from the HMCK in the observed karyotypes. The first number indicates the number of chromosomes, while deviations are listed after a comma, with “−MMIn” and “+MMIn” indicating the loss or gain of one copy of chromosome “n” compared to the HMCK, respectively. For example, the most frequent 2*n* = 9 karyotype consisted of one unpaired largest metacentric chromosome (chromosome #1, MMI1), one pair of large metacentrics (chromosome #2, MMI2), and three pairs of small metacentrics (MMI3–MMI5) (Figure 1c and Figure 3a), and it is thus designated as 9,−MMI1. All chromosome morphometry was carried out on the metaphase plates of the specimens showing this most frequent 9,−MMI1 karyotype, and the results are shown in Table 2.

In 18 specimens (34.6%), chromosome numbers deviated from 2*n* = 9, varying from 2*n* = 7 (one large and six small chromosomes) to 2*n* = 14 (six large and eight small chromosomes). In some of them, structurally rearranged chromosomes were found (Figure 3b–f, Table 1 for a complete list of karyotypes see Table S1). In karyotypes of seven specimens (13.5%), one very small chromosome was revealed. We think these chromosomes can be referred to as B chromosomes (i.e., the karyotype is 10,−MMI1,+1B). Among the remaining 11 specimens, the chromosome number variation occurred in the small chromosomes (from seven to nine copies) and/or the large chromosomes (from one to six copies of MMI1 and/or MMI2). No mosaics were revealed among the karyotyped specimens.

After one year of cultivation under standard laboratory conditions (~13 generations) karyotyping was performed on 100 randomly chosen worms. Although the worms did not show any obvious abnormalities in terms of behavior (e.g., swimming in circles) or morphology (i.e., carrying cysts or other malformations), the analysis revealed increased karyotype diversity. Of the analyzed worms, only 20% showed karyotype 9,−MMI1, while 26% showed the HMCK (i.e., 2*n* = 10: two copies of MMI1, two copies of chromosome MMI2, and six small chromosomes, MMI3–MMI5). The remaining specimens (54%) showed a range of different karyotypes, including numerical variation from five (two large chromosomes similar in size and three small chromosomes) up to 23 (seven large and 16 small chromosomes). Structurally rearranged chromosomes were also revealed in the analyzed specimens (Figure 3g–l, Table 1 and Table S1).

To be sure that we counted the chromosome numbers correctly, we carefully analyzed all available good-quality metaphase spreads (≥10 spreads for each specimen). For example, 17 metaphase spreads for the specimen with the 2*n* = 5 karyotype were analyzed (Figure 3h), and all of them contained five chromosomes showing identical morphology (two large and three small metacentric chromosomes). In most specimens, all analyzed spreads contained identical chromosome sets. In only two specimens, the spreads contained different numbers of chromosomes. In both worms, five analyzed spreads contained nine chromosomes (three large chromosomes and six small metacentrics), while five other spreads contained eight chromosomes (two large chromosomes and six small metacentrics).

#### 2.1.4. *Macrostomum cliftonensis*

**Figure 4:**
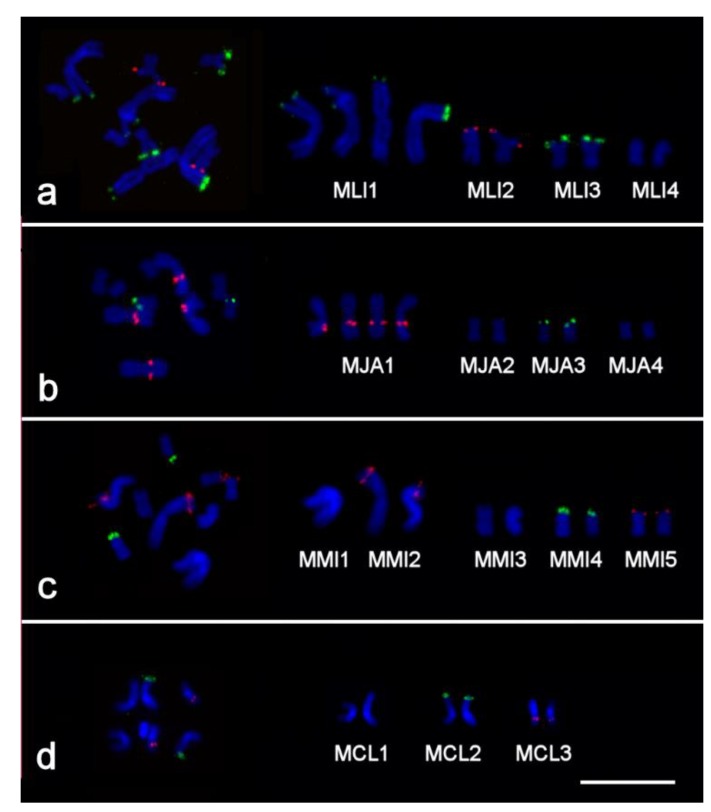
Two-color fluorescent in situ hybridization (FISH) with probes specific to 28S rDNA (green) and 5S rDNA (red) on chromosomes of four *Macrostomum* species: (**a**) inbred DV1/10 line of *M. lignano* and outbred cultures of (**b**) *M. janickei*, (**c**) *M. mirumnovem,* and (**d**) *M. cliftonensis*. Chromosomes were counter-stained with DAPI (blue). Scale bar 10 µm.

The karyotype of *M. cliftonensis* consists of three pairs of similar-sized metacentric chromosomes (2*n* = 6) that are comparable in size to the small chromosomes in the other three species (Figure 1d). We did not find any numerical or structural chromosomal abnormalities in 100 karyotyped specimens (Table 1), suggesting that the karyotype of this species appears to be stable after one year of cultivation under the standard laboratory conditions. The only variation revealed during *M. cliftonensis* karyotyping was a secondary constriction in one homolog of the largest chromosome, and this unusual chromosome was observed in only two specimens. In one of them, it was found in the only metaphase plate examined (in 2017) (Figure 2i). In another specimen (in 2018) it was present in two metaphase plates. We think that the appearance of this secondary constriction is unlikely to be linked to the activation of the nucleolus organizer region (NOR), since the 28S rDNA region, which is usually associated with the NOR, is located in the terminal region of the MCL2 chromosome of *M. cliftonensis* (see Figure 4d).

### 2.2. Clusters of 5S rDNA, 28S rDNA, and Telomeric Repeats in Chromosomes of Studied Species

In specimens of the DV1/10 line of *M. lignano*, the 28S rDNA clusters were observed in the terminal regions of chromosomes MLI1 and MLI3 (Figure 4a) and showed variation in size, as described earlier for the DV1 line of that species [36]. The 5S rDNA cluster was found in the terminal region of chromosome MLI2 (Figure 4a), also as previously reported [39]. In *M. janickei*, the 28S rDNA clusters were localized in the terminal region of chromosome MJA3 (also as reported earlier [38]), while the 5S rDNA cluster was localized in the proximal region of the q-arm of chromosome MJA1 (Figure 4b).

In *M. mirumnovem*, the 5S rDNA clusters were found in the terminal region of chromosome MMI5 and in the distal part of the p-arm of chromosome MMI2 (Figure 4c), the latter larger than the former. The 28S rDNA cluster was localized in the terminal region of the p-arm of chromosome MMI4 (Figure 4c). Finally, in *M. cliftonensis*, the 28S rDNA cluster was observed at the end of the p-arm of chromosome MCL2, while the 5S rDNA cluster was localized in the distal part of the q-arm of chromosome MCL3 (Figure 4d).

**Figure 5:**
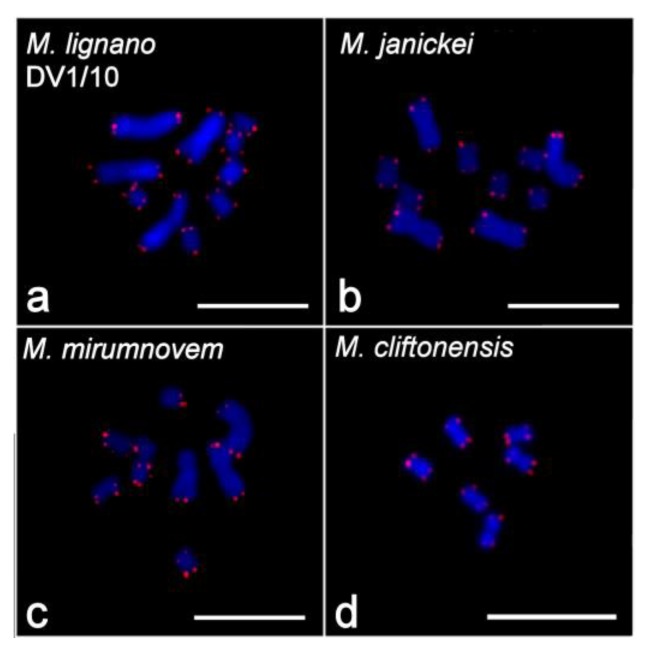
FISH with labeled telomeric DNA (red) on chromosomes of four *Macrostomum* species: (**a**) *M. lignano*, (**b**) *M. janickei*, (**c**) *M. mirumnovem*, and (**d**) *M. cliftonensis*. Chromosomes were counter-stained with DAPI (blue). Scale bar 10 µm.

In all four studied *Macrostomum* species, clusters of telomeric DNA repeats were observed at the ends of the chromosomes (Figure 5), and no additional interstitial clusters of telomeric DNA were observed in any specimens. The results of the fluorescent in situ hybridization (FISH) analysis in *M. lignano* and *M. janickei* after additional cultivation time in the laboratory were identical to those obtained earlier [36]. The data on clusters of telomeric repeats in *M. mirumnovem* and *M. cliftonensis* also appeared to be the same after one year of cultivation as at the beginning. However, we should note that we currently have no information on the distribution of telomeric DNA repeats in the B chromosomes of *M. mirumnovem*, since this would require additional FISH experiments on specimens with such B chromosomes.

### 2.3. FISH with Microdissected DNA Probes on Macrostomum Chromosomes

#### 2.3.1. Cross-Species In Situ Hybridization

**Figure 6:**
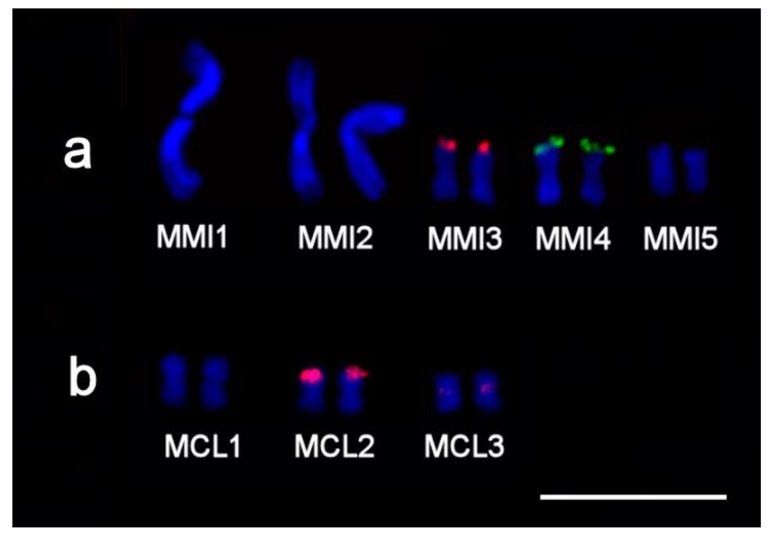
FISH with microdissected DNA probes derived from *M. lignano* chromosomes (*Mli2*, green signal; *Mli3_4*, red signal) on metaphase chromosomes of (**a**) *M. mirumnovem* and (**b**) *M. cliftonensis.* Chromosomes were counter-stained with DAPI (blue signal). Scale bar 10 µm.

We previously showed that microdissected DNA probes derived from chromosomes of *M. lignano* (namely *Mli2* and *Mli3_4*) provide specific FISH painting patterns of both *M. lignano* and *M. janickei* chromosomes [38]. Moreover, no difference was observed in the intensity of the FISH signals on paralogous chromosome regions of these two species (Figure S2), suggesting recent divergence between them. When we applied the same DNA probes to chromosomes of *M. cliftonensis* and *M. mirumnovem*, however, no specific fluorescence signals marking extended chromosome regions were revealed (Figure 6a,b), while in control experiments on the *M. lignano* and *M. janickei* chromosomes they again specifically painted the corresponding chromosomes and chromosome regions (as shown in Figure S2). We should note that according to a recent molecular phylogenetic analysis, all the species studied here belong to the same subclade of the genus *Macrostomum*, and they are therefore fairly closely related compared to most other *Macrostomum* species [34,40] (see also Figure S1). However, it appears that the phylogenetic separation between the respectively more closely related *M. mirumnovem/M. cliftonensis* and *M. lignano*/*M. janickei* species pairs permitted the accumulation of sufficient changes in unique DNA sequences to prevent specific painting of paralogous chromosome regions by FISH when using microdissected DNA probes derived from *M. lignano.*

Nevertheless, FISH with these microdissected *M. lignano* DNA probes gave intensive signals in the distal parts of some chromosomes of both *M. cliftonensis* and *M. mirumnovem*, and thus allowed clusters of DNA repeats that are apparently present in the used DNA probes to be revealed. Specifically, FISH with *Mli3_4* (which includes the MLI3 chromosome and is thus expected to include the 28S rDNA cluster) gave a strong signal in the distal part of the p-arm of chromosome MMI3 in *M. mirumnovem* and the distal part of the p-arm of chromosome MCL2 in *M. cliftonensis* (Figure 6a,b), while FISH with *Mli2* (which is expected to include the 5S rDNA cluster) gave a strong signal only in the distal part of the p-arm of chromosome MMI4 in *M. mirumnovem* (Figure 6a). The distal region of MMI4 was simultaneously painted with the DNA probes 28S rDNA and *Mli3_4*, which means that it was additionally enriched with other clustered DNA repeats. Regions containing clusters of rDNA genes are usually flanked by C-positive regions containing repetitive DNA. Probably, FISH with the DNA probes *Mli2* and *Mli3_4* revealed such regions enriched in repeats.

#### 2.3.2. Microdissected Region-Specific DNA Probes Obtained from Large Chromosomes of *M. mirumnovem*

According to its chromosome morphology, the *M. mirumnovem* karyotype was similar to the karyotypes of some specimens from the DV1 culture of *M. lignano* [36]. The only difference between them was the increased size of one of the large metacentrics. This similarity prompted us to apply an approach to the analysis of *M. mirumnovem* that previously appeared to be successful in the study of *M. lignano* chromosomes [38,39]. Specifically, we prepared microdissected DNA probes from the proximal region (*Mmi2med*) and from combined distal regions (*Mmi2dist*) of the large paired metacentric chromosome (MMI2) and performed FISH on metaphase chromosomes of *M. mirumnovem*. MMI2 of *M. mirumnovem* was identified based on its size, and 15 copies of the relevant proximal and distal chromosome regions were collected and DNA probes were generated with standard techniques [38,39].

**Figure 7:**
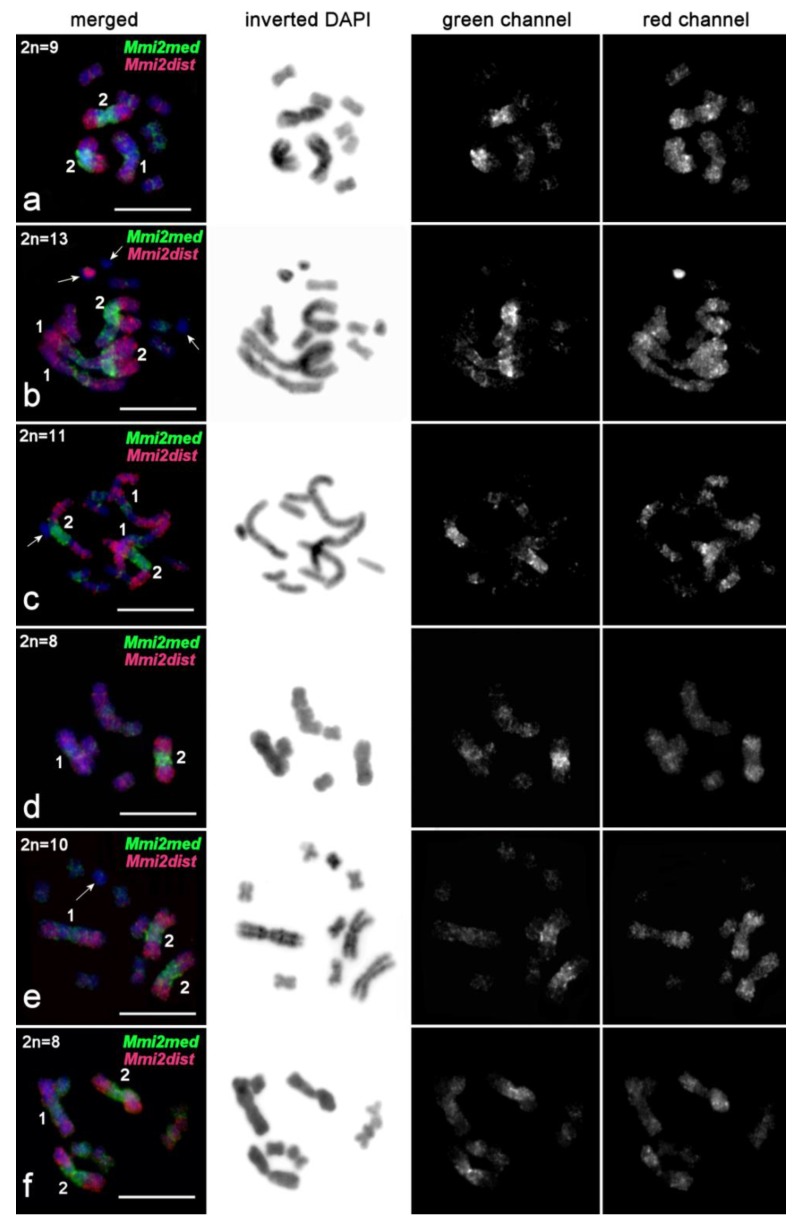
FISH with microdissected region-specific DNA probes derived from chromosome MMI2 of *M. mirumnovem* on chromosomes of the original species. Separate channels (*Mmi2med*, green signal; *Mmi2dist*, red signal) and merged images are shown. FISH was performed on metaphase plates with different chromosome numbers. B chromosomes are shown by arrows. (**a**) 9,−MMI1; (**b**) 13,+3B (four large and six small metacentrics, and three B chromosomes); (**c**) 11,+1B (four large and six small metacentrics, and one B chromosome); (**d**) 8,−MMI1,−MMI2 (two large and six small metacentrics); (**e**) 10,–MMI1,+1B (three large and six small metacentrics, and one B chromosome; (**f**) 8,−MMI1,−MMI4 (three large, and five small metacentrics). Chromosomes were counterstained with DAPI (blue). Scale bar 10 µm.

Two-color FISH with *Mmi2med* and *Mmi2dist* on the *M. mirumnovem* chromosomesintensively painted the original regions of MMI2 (Figure 7, Table S2). Moreover, these DNA probes also slightly painted the medial and distal regions of MMI1 and gave specific signals of lower intensity on some of the small metacentrics (Figure 7, Table S2). The FISH signal on the small metacentrics of specimens with the 9,–MMI1 karyotype was nonhomogeneous. The *Mmi2med* probe mostly gave signals on MMI5 and in the pericentric regions of the other small metacentrics, while the *Mmi2dist* probe weakly and unevenly painted chromosomes MMI3 and MMI4 (Figure 7, Table S2).

In some specimens, besides chromosomes corresponding to the 9,−MMI1 *M. mirumnovem* karyotype (according to their morphology), additional small chromosomes were observed. Some of these chromosomes showed a very intensive FISH signal (Figure 7b), while others showed no FISH signal at all (Figure 7b,c,e), and they probably consisted mainly of DNA repeats that either are or are not present in the probes used. In the following, we refer to these chromosomes as Bs.

To interpret the painting patterns on large chromosomes, we should take into account the possible contamination of the *Mmi2med* and *Mmi2dist* DNA probes with DNA from chromosome MMI1. Specifically, we cannot completely exclude errors in the identification of chromosome MMI2 during microdissection, since the size difference between MMI1 and MMI2 is modest. Erroneous collection of chromosomal material could potentially provide painting patterns on MMI1 similar to those on MMI2, even in the absence of homology between chromosomes MMI1 and MMI2. To exclude the possibility of any contamination, in further experiments, we generated microdissected DNA probes from single copies of each chromosome. As we outline next, FISH with DNA probes derived from single-copy small chromosomes confirmed the results obtained with the *Mmi2med* and *Mmi2dist* probes, with both indicating partial homology between MMI1 and MMI2.

#### 2.3.3. FISH with Single-Copy Microdissected DNA Probes Derived from Chromosomes of *M. mirumnovem*

**Figure 8:**
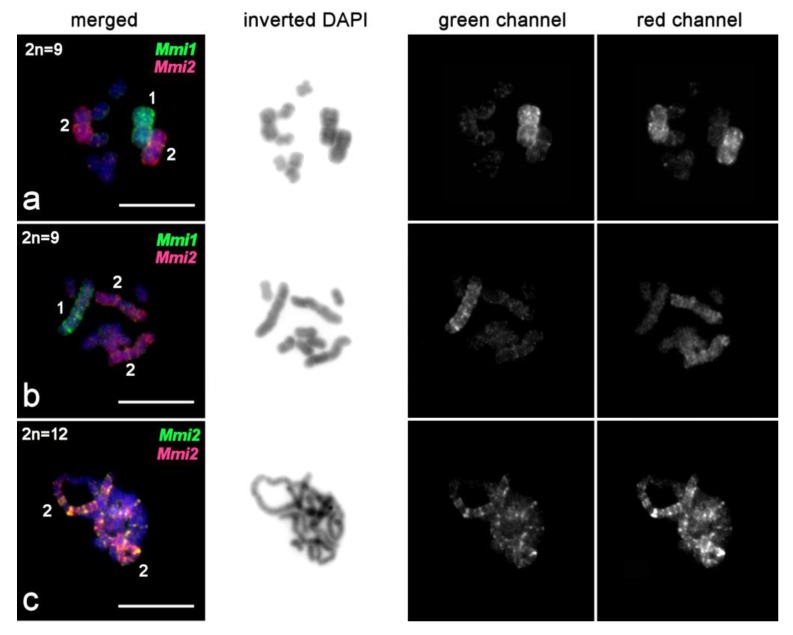
Chromosome painting with single-copy DNA probes on chromosomes of *M. mirumnovem*. (**a**,**b**) FISH with DNA probes derived from chromosomes 1 (green signal) and 2 (red signal); (**c**) FISH with two DNA probes derived from different homologs of MMI2 (green and red signals). Chromosomes were counterstained with DAPI (blue). Scale bar 10 µm.

**Figure 9:**
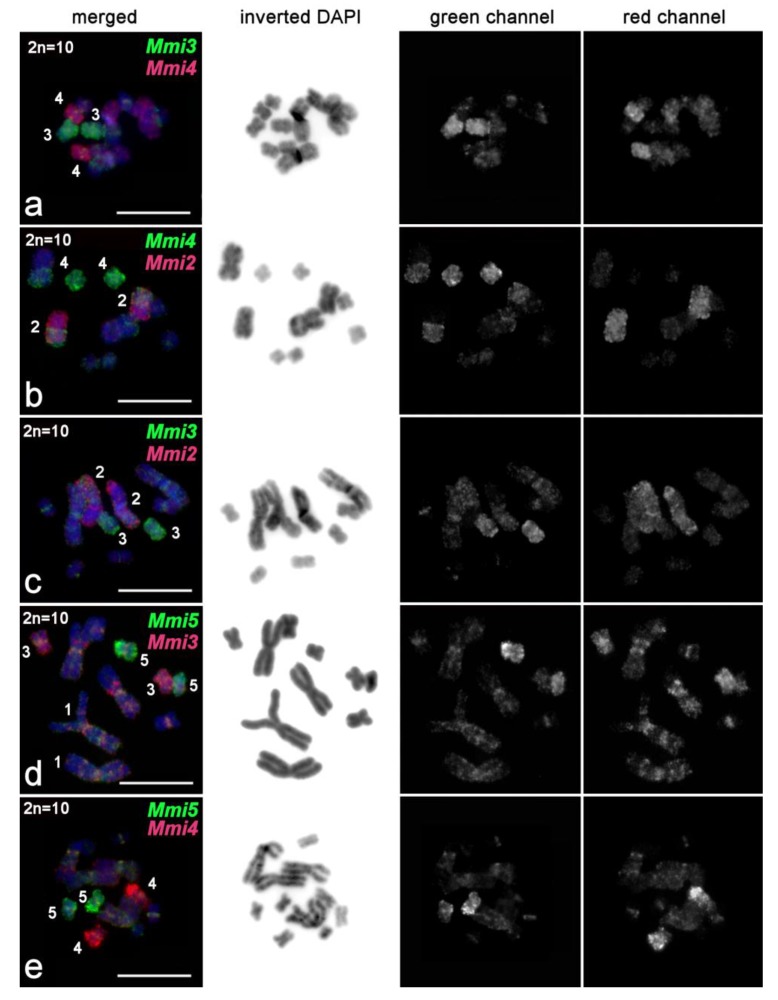
Chromosome painting with single-copy DNA probes on chromosomes of *M. mirumnovem*. FISH with (**a**) *Mmi3* (green) and *Mmi4* (red) probes; (**b**) *Mmi4* (green) and *Mmi2* (red) probes; (**c**) *Mmi3* (green) and *Mmi2* (red) probes; (**d**) *Mmi3* (red) and *Mmi5* (green) probes; (**e**) *Mmi4* (red) and *Mmi5* (green) probes. Chromosomes were counterstained with DAPI (blue).

DNA probes were generated from single copies of each chromosome collected from a 9,–MMI1 metaphase spread (and probes from homologous chromosomes pooled after verification; see Materials and Methods). FISH with these DNA probes provided a specific signal on the original chromosome (Figure 8 and Figure 9). We should note, however, that the applied modification of the chromosomal in situ suppression (CISS) hybridization protocol did not eliminate FISH signals derived from clustered repeats providing signals of different intensity (from low to high). Such high-intensity signals in chromosome regions enriched for repetitive DNA were revealed in both pericentromeric chromosome regions and chromosome arms. Especially numerous high-intensity signals were observed in the arms of MMI1 and MMI2. On highly condensed chromosomes MMI1 and MMI2, the presence of regions enriched for repetitive DNA homologous to labeled DNA could increase the FISH signal that resulted in almost even painting patterns, while on low condensed chromosomes, intensively painted chromosome regions enriched with such repeats alternated with less intensively painted regions, mostly containing unique DNA sequences and/or repeats showing no homology to the labeled DNA probe (Figure 8c).

We should note that FISH with *Mmi1* and *Mmi2* mostly revealed regions enriched for repetitive DNA on the original chromosomes (Figure 8), providing almost no FISH signal on other chromosomes, while FISH with *Mmi2med* and *Mmi2dist* also painted regions in the other chromosomes. This can probably be explained by the generation of *Mmi1* and the *Mmi2* from single copies of the corresponding chromosomes, while the other probes were generated from pooled chromosome samples. FISH with *Mmi1* and *Mmi2* probes gave intensive signals in regions enriched for repeat clusters that masked less intensive specific signals on other chromosomes. *Mmi2med* and *Mmi2dist* were generated from 15 copies of chromosome regions, and FISH with these probes provided more intensive signals on paralogous chromosome regions.

FISH with the *Mmi3*, *Mmi4*, and *Mmi5* DNA probes painted the original chromosomes evenly and additionally provided signals of lower intensity in regions of the large chromosomes MMI1 and MMI2 (Figure 9). These DNA probes provided different painting patterns on the large chromosomes. *Mmi4* painted mostly one arm in both MMI1 and MMI2 (Figure 9b), a little more intensively in MMI2 than MMI1 (Figure 9b). FISH with the *Mmi3* and *Mmi5* DNA probes painted small alternating regions in both large chromosomes (Figure 9d, Table S2). It is not easy to differentiate between FISH signals coming from regions enriched with repeat clusters compared to ordinary euchromatic regions, but a comparison of chromosomes of different condensation levels allows us to suggest that DNA probes derived from MMI3–MMI5 usually did not intensively paint the regions enriched with repetitive DNA (Figure 9c). In addition, based on FISH results obtained with the *Mmi3*, *Mmi4*, and *Mmi5* DNA probes, we could not differentiate small paralogous euchromatic regions and regions enriched with repetitive DNA in the MMI1 and MMI2 chromosomes. Taking the FISH results with all DNA probes together, we can describe only the distribution of small regions that were marked by the FISH signals from different DNA probes (Table S2). In addition to the pericentromeric regions, the repeat-enriched regions were distributed mostly along large chromosomes, and many of them were chromosome-specific. Two-color FISH with single-copy chromosome-specific DNA probes derived from two homologs of MMI2 showed that copy number variation of DNA repeats in various copies of chromosome MMI2 could be present (Figure 8c).

#### 2.3.4. FISH Analysis of Copy Number Variation of Large Chromosomes and Presence of B Chromosomes in *M. mirumnovem*

FISH using the *Mmi1* and *Mmi2* DNA probes allowed us to detect copy number variations of the MMI1 and MMI2 chromosomes in specimens with different karyotypes consisting of eight to 13 chromosomes (Table S3). In specimens studied in 2018, the number of copies of MMI1 varied from one to two, while MMI2 could be present in one to three copies. As B chromosomes, we determined small chromosomes (usually smaller than MMI3–MMI5) showing either a very intensive FISH signal or no FISH signal at all. We suggest that these patterns of chromosome painting are indicative of extreme enrichment of these chromosomes for repetitive DNA. The copy numbers of B chromosomes in the studied specimens ranged from zero to three and were stable in the analyzed specimens (Table S3). Additional studies of more specimens with B chromosomes are required for detection and description of the stability of these chromosomes and their morphotypes.

## 3. Discussion

The karyotypes of the *Macrostomum* species studied to date can be divided into three groups based on their chromosome numbers and morphology: (i) many species have a 2*n* = 6 chromosome set; (ii) at least one species, *M. hustedi*, has the double chromosome number, 2*n* = 12; and (iii) three species have asymmetric karyotypes and intermediate chromosome numbers, ranging from 2*n* = 8 to 2*n* = 10 [35,36], taking the current results into account. Of these, the 2*n* = 6 karyotype has previously been considered to be the basal pattern for the genus *Macrostomum* [35], while the species with 2*n* = 12 could have resulted from a WGD. However, since *M. hustedi* has not yet been placed phylogenetically, we will not discuss it further here. Recently, molecular cytogenetic studies of two closely related *Macrostomum* species with intermediate chromosome numbers and asymmetric karyotypes (*M. lignano* and *M. janickei*) showed that they had undergone WGD by autotetraploidy, followed by fusion of a haploid ancestral chromosome set, leading to the formation of a large metacentric chromosome [39]. In the current study, we described an additional *Macrostomum* species with an asymmetric karyotype, *M. mirumnovem*, which appeared to be similar to the earlier described *Macrostomum* species. Our results, based on chromosome microdissection and FISH, show that *M. mirumnovem* also underwent WGD accompanied by similar chromosome fusions. However, we observed numerous species-specific particularities in the karyotypes of *M. mirumnovem* specimens cultured in the laboratory, which are quite distinct from the observations made in the other species. Specifically, the studied specimens showed extremely high levels of karyotypic instability, including variations in chromosome number, structural chromosome rearrangements, distribution and amplification of repetitive DNA regions, and the appearance B chromosomes. In the following, we first discuss the evidence for these observations in *M. mirumnovem*, and then evaluate different evolutionary scenarios that could help to account for the observed patterns.

### 3.1. Differentiation of Large Chromosomes and Origin of B Chromosomes in M. mirumnovem

In contrast to the large chromosomes of *M. lignano* and *M. janickei*, which were almost identical in size, morphology, and painting pattern [38], the large chromosomes of *M. mirumnovem* appeared to be different. For example, MMI1 is not only larger than MMI2, but it also showed different painting patterns after FISH with microdissected DNA probes. Specifically, the FISH results with DNA probes derived from the small metacentrics suggest that initially the large chromosomes of *M. mirumnovem* were likely formed by fusion of the same chromosomes and their differentiation, then occurred after chromosome fusion, in that the MMI1 and MMI2 chromosomes showed different regions enriched for various repeats. We suggest a two-step scenario for the differentiation of the large chromosomes in *M. mirumnovem*. In the first step after chromosome fusion, the independent settling of different transposable elements (TEs) occurred along different chromosome copies of the large chromosome. Then only TEs or TEs together with adjacent DNA sequences were amplified. As a result, numerous repeat clusters or regions enriched for different repeats were distributed along the chromosome, leading to different copies of the original chromosome [41,42]. The next step likely included the loss of various euchromatic regions located between the repeat clusters or regions enriched for repeats.

This scenario is in good agreement with the FISH results obtained with microdissected DNA probes. The DNA probes *Mmi3–Mmi5*, which originated from the small chromosomes, painted regions on the large chromosome MMI1 more intensively than did the DNA probe *Mmi2*, which originated from the other large chromosome, MMI2. According to our suggestion, small metacentrics contain all chromosome regions that are present in the corresponding parts of MMI1, while some of these regions were lost from MMI2. A similar phenomenon of a loss of euchromatic regions between small C-positive regions enriched with repeats was observed in the evolution of the neo-Y chromosome in some grasshopper species from the Pamphagidae family [43]. Small C-positive regions were distributed along the neo-Y chromosome in the initial stage of its evolution, and in the next stage of neo-Y degradation they were fused after the loss of euchromatic regions located between them [43]. In another grasshopper species, *Eyprepocnemis plorans*, numerous DNA repeat clusters were found to be distributed along the autosome [44]. It was suggested that deletions of euchromatic regions located between the clusters of repeats could lead to the autosome degradation up to the small heterochromatic B chromosome.

In *M. mirumnovem,* besides numerous regions in the large chromosomes that were extremely enriched with DNA repeats, we also observed B chromosomes. FISH with the DNA probes *Mmi1* and *Mmi2* painted some B chromosomes differently. For example, one of the Bs was painted intensively (Figure 7b), while another one showed no FISH signal at all (Figure 7c,e). We suppose that the B chromosomes observed in *M. mirumnovem* arose due to different mechanisms: (i) formation of a small supernumerary chromosome containing mostly centromeric and pericentromeric regions with subsequent amplification of repetitive DNA, or (ii) loss of euchromatic regions in the original large chromosome. We should note that in the current study, the copy number of B chromosomes in *M. mirumnovem* might be underestimated, since the morphology of some B chromosomes may be similar to that of A chromosomes. In the future, such Bs could be omitted from the analysis using routine chromosome staining. However, we should note that the revealed B chromosomes open a new possibility for analysis of amplified DNA repeats in *M. mirumnovem*. Microdissected DNA libraries can be generated from B chromosomes and then used for NGS sequencing, as was done previously for the Bs in some species of different taxa [45,46,47]. The study of B chromosomes in *M. mirumnovem*, including the generation of microdissected libraries and their sequencing is in progress.

### 3.2. Possible Reasons for Karyotypic Instability in Post-WGD Macrostomum Species

The high level of karyotypic instability in the post-WGD *Macrostomum* species, especially in *M. mirumnovem*, is one of the most exciting findings of the present study. However, we should note that most of the karyotyped specimens were taken from laboratory lines and cultures, and it seems conceivable that at least part of the observed variation could be associated with the laboratory conditions. However, we previously assessed karyotype variation in specimens of *M. lignano* and *M. janickei* collected directly from natural populations [36]. Among 122 specimens of *M. lignano* and 22 specimens of *M. janickei,* this revealed two (1.6%) and four (18%) specimens, respectively, with abnormal karyotypes [36]. These data thus clearly indicate that appreciable levels of karyotype instability also occur in natural populations, particularly *M. janickei.*

Although *M. cliftonensis* also showed a stable 2*n* = 6 karyotype after one-year cultivation (as did other two species for which long-term cultures were maintained, *M. hystrix* and *M. spirale* [36]), we cannot exclude the possible impact of laboratory cultivation on the stability of chromosome numbers in the studied post-WGD species. It is possible that the level of karyotype instability in natural populations is lower than what we observe in laboratory cultures. The increased karyotype diversity in the laboratory cultures could arise due to weaker selection against odd karyotypes under the likely more favorable laboratory culture conditions. Indeed, in all studied post-WGD *Macrostomum* species, the karyotypic instability appeared to increase after long-term cultivation under the standard laboratory conditions. Unfortunately, we do not have detailed data on the level of karyotypic instability in natural populations of *M. lignano*, *M. janickei,* and *M. mirumnovem*, so the question of possible induction of karyotypic instability in laboratory cultures remains open and an interesting direction for further studies.

### 3.3. Comparative Cytogenetic Analysis of Chromosomes of Macrostomum Species

Considering the results of karyotyping performed on the chromosomes of the studied post-WGD *Macrostomum* species, their karyotypes look quite similar, showing nearly the same chromosome number and morphology and karyotypic instability. Moreover, FISH with microdissected DNA probes derived from the *M. lignano* chromosomes revealed a high level of DNA homology with *M. janickei*, while the DNA divergence between *M. lignano* and *M. mirumnovem*/*M. cliftonensis* appeared to be significant. Specifically, FISH with microdissected DNA probes derived from the *M. lignano* chromosomes revealed no orthologous chromosome regions. However, these DNA probes gave a strong local FISH signal in two chromosome regions in both *M. mirumnovem* and *M. cliftonensis*. Neither of these regions contained gene clusters containing 5S or 28S rDNA genes. Instead, they were probably enriched in repeats that are homologous to DNA present in the corresponding DNA probe. The identification of such regions in the chromosomes of both *M. mirumnovem* and *M. cliftonensis* is a very intriguing finding in the obtained data, since it may suggest a relatively recent divergence between these species. Indeed, the current phylogenetic tree of these related species in the genus *Macrostomum* suggests that *M. mirumnovem* and *M. cliftonensis* are likely fairly closely related (Figure S1). Unfortunately, it is currently not possible to reach a firm conclusion about the specific chromosome rearrangements that have led to differentiation of the chromosomes of the *M. lignano*/*M. janickei* lineage and the other studied species. To clarify this question, FISH experiments using the chromosome-specific DNA probes generated from many chromosome copies or labeled cloned DNA fragments are required.

### 3.4. Hypothetical Scenarios of WGD in the M. lignano/M. janickei and M. mirumnovem Lineages

Based on the obtained data, several scenarios for genome evolution among the studied species can be suggested. In Scenario I we assume a common WGD event for the three post-WGD species as the first stage (Figure S3a), followed by karyotype reorganization events in the subsequent genome evolution: (i) the fusion of a haploid chromosome set into one large metacentric chromosome, (ii) an increase in the copy number of these large chromosome(s), and (iii) karyotypic instability. Under this scenario, the formation of a large chromosome would likely have occurred in the common ancestor of a hypothetical clade containing the *M. lignano*/*M. janickei* and *M. mirumnovem* lineages, followed by a divergence of the duplicated and rearranged genome between these lineages (Figure S3a). The main argument against Scenario I is that it is contradicted by the molecular phylogenetic interrelationships between the studied *Macrostomum* species (Figure S1). These data supports a clade containing *M. lignano* (from Italy and Greece) and *M. janickei* (from France) with fairly high confidence, and these close sibling species have recently been shown to be capable of producing viable hybrid progeny [48]. The phylogenetic tree further identifies a somewhat less well supported clade containing *M. mirumnovem* (from Victoria, Australia) and *M. cliftonensis* (from Western Australia) [34] (Figure S1). The species *M. cliftonensis* and *M. mirumnovem* are more distantly related to *M. lignano*, but they may also represent fairly closely related species (Figure S1). The topology of this molecular phylogeny is also in good agreement with our results of the cross-species FISH experiments using microdissected DNA probes (see previous sections).

Moreover, based on the presence of large paralogous chromosome regions in the *M. lignano* and *M. janickei* karyotypes (Figure S2), we previously suggested that genome doubling in the *M. lignano*/*M. janickei* lineage was a result of autotetraploidization [38], which under Scenario I would also underlie the *M. mirumnovem* karyotype. Moreover, under Scenario I, the WGD event would need to have occurred a long time ago to allow for the observed genetic divergence between the *M. lignano/M. janickei* and *M. mirumnovem* lineages to emerge, but subsequently leading to quite different patterns of genome evolution in the two lineages. According to this scenario, karyotypic instability in *M. mirumnovem* might then have appeared as a result of a more recent event, for example, the acquisition of a new TE incorporation (e.g., through introgression) after separating from the *M. lignano/M. janickei* lineage and its subsequent settling across the *M. mirumnovem* genome. It is also possible that the prominent karyotypic instability was induced by cultivation under laboratory conditions. However, the distribution of the newly formed regions enriched with repeats seems to be restricted mostly to the large chromosomes. For these reasons, the suggestion that *M. mirumnovem* was formed through another WGD compared to *M. lignano* and *M. janickei* seems to be more plausible.

Future molecular phylogenetic studies on the genus *Macrostomum* could potentially alter the topology of the current phylogenetic tree, which is currently based solely on a partial sequence of the mitochondrial COI gene. However, it currently seems unlikely that a new topology will support the hypothesis of a single common WGD event, since more ongoing phylogenomic analyses of the genus *Macrostomum* also support the interpretation of multiple WGD events (L. Schärer, personal observation).

Therefore, we favor a second hypothetical scenario, Scenario II, which assumes two independent WGD events (Figure S3b,c), one occurring in the *M. mirumnovem* lineage and the other in the *M. lignano*/*M. janickei* lineage (i.e., a scenario that agrees with the current phylogeny). Moreover, within Scenario II it is possible to consider two subscenarios that include different mechanisms of WGD (Figure S3b,c). Under Scenario IIa, the ancestral genomes are assumed to have undergone separate WGDs through autotetraploidization in both the *M. lignano*/*M. janickei* and *M. mirumnovem* lineages (Figure S3b). The observed differences in the level of karyotypic instability in the two phylogenetic lineages could then potentially be explained by different levels of TE activation after the independent WGD events and/or by a different age of the WGD within each clade. Under Scenario IIb, we also assume two independent WGD events, but we further assume that they involved different mechanisms (Figure S3c). Specifically, on the one hand, we still assume a WGD by autotetraploidization in the *M. lignano/M. janickei* lineage without strong TE activation, leading to the emergence of species with more stable karyotype and large paralogous regions in their chromosomes. On the other hand, we assume a WGD by allopolyploidization in the *M. mirumnovem* lineage, which was then accompanied by a burst of TE activity that led to the emergence of a species with a higher level of instability. This process could help to explain the formation of numerous large chromosome-specific regions enriched for DNA repeats, as well as B chromosomes (Figure 8 and Figure 9). Unfortunately, our findings currently provide only indirect evidence in favor of an allopolyploid origin of the *M. mirumnovem* lineage. As an additional argument in support of this allopolyploid hypothesis, we can mention that the small chromosomes in the *M. mirumnovem* karyotype do not contain numerous regions enriched for repeats, with such repeats being restricted mostly to the large chromosomes. Similar differences between parental subgenomes have previously been described as subgenome dominance in some allopolyploid plants [49]. With respect to the hypothesis of WGD through autopolyploidization in the *M. lignano/M. janickei* lineage, we should acknowledge that it is difficult to distinguish autopolyploidization from genome duplication resulting from the fusion of unreduced gametes derived from individuals of closely related species.

One potential difficulty with Scenario II is that it suggests that the two different WGD events were followed by very similar independent karyotype reorganization processes. While this suggestion looks like an argument against the hypothesis of independent WGD events, numerous chromosome fusions have previously been described in the karyotype evolution of many taxa, for instance, the mole vole, *Ellobius tancrei*; the common shrew, *Sorex araneus*; and other mammalian species [50,51,52,53]. They led to the formation of chromosome races that differed with respect to the combination of fused chromosomes (“Robertsonian fans”) [50,51] or to a strong reduction in chromosome number, as observed in the evolution of small deer of the genus *Muntiacus* [52,54,55]. This may suggest that it is not so unlikely that we are dealing with independent chromosome fusions in the genome evolution of the *M. mirumnovem* and *M. lignano/M. janickei* lineages.

In conclusion, we would like to note that the post-WGD *Macrostomum* species (*M. lignano*, *M. janickei*, and *M. mirumnovem*) analyzed in this study represent a very promising model system for studying the mechanisms and regularities of karyotype and genome evolution after a recent WGD in animals. Moreover, it appears possible that the level of karyotypic instability was significantly increased during laboratory cultivation, which might allow us to observe karyotype and genome reorganization over short time frames, with karyotyping of relatively small numbers of specimens. For more detailed analyses of karyotype evolution in these species, it will be necessary to combine molecular karyotyping of specimens from laboratory cultures and different natural populations, including comparative cytogenetics of specimens from different populations and localities. Further comparative karyotyping of additional *Macrostomum* species, including some species with the mentioned 2*n* = 12 karyotype, represents a promising direction for further studies.

## 4. Materials and Methods

### 4.1. Study Organisms

The specimens of the four *Macrostomum* flatworm species analyzed in this study were derived from natural populations and are currently being maintained as inbred lines or outbred cultures under standard laboratory conditions [56,57]. The specimens of *M. cliftonensis* Schärer & Brand 2019 (previously referred to as *Macrostomum* sp. 84) and *M. mirumnovem* Schärer & Brand 2019 (previously referred to as *Macrostomum* sp. 94) were collected in 2017 from populations of Lake Clifton (Western Australia) and Port Philipp Bay (Victoria), respectively [34]. The specimens of *M. janickei* Schärer 2019 (previously referred to as *Macrostomum* sp. 8) were collected in 2014 in Palavas-les-Flots, Southern France [34,36]. Finally, we also used specimens of *M. lignano* Ladurner, Schärer, Salvenmoser & Rieger 2005 that were derived from DV1/10 [38,39], a subline of the commonly used DV1 inbred line [57,58,59]. Specifically, DV1/10 was established in 2015, by starting a subline of DV1 from individuals with a 2*n* = 10 chromosome set, with the aim of establishing a potentially stable line with the 2*n* = 10 karyotype (i.e., four large and six small metacentric chromosomes), including four identical copies of the large chromosome [38]. Since then DV1/10 has been regularly karyotyped to check its stability.

### 4.2. Metaphase Chromosome Preparation

Chromosome slides obtained from individual worms were prepared according to the previously described single-worm karyotyping technique [36]. Unless otherwise stated, at least 10 metaphase plates were analyzed, in order to permit checking for mosaicism. The karyotyping of *M. mirumnovem* was performed twice, once after 3 months of cultivation under laboratory conditions and once after 12 months. Karyotypes of the remaining three *Macrostomum* species (*M. lignano* DV1/10, *M. janickei*, and *M. cliftonensis*) were analyzed at least twice (for detailed information see Table 1). Chromosome slides for metaphase chromosome microdissection of *M. mirumnovem* and FISH experiments on chromosomes of *M. lignano*, *M. janickei*, *M. cliftonensis, M. mirumnovem* were prepared from batches of 100 specimens according to the cell-suspension technique [36].

### 4.3. Chromosome Staining

Metaphase chromosomes on slides were stained with 4′,6-diamidino-2-phenylindole solution (DAPI) dissolved in VECTASHIELD^®^ Antifade Mounting Medium (Vector Laboratories Inc., Burlingame, CA, USA) according to standard protocol. For metaphase chromosome microdissection, chromosomes on coverslips (60 mm × 24 mm × 0.17 mm) were stained with 0.1% Giemsa solution (Sigma-Aldrich, St. Louis, MO, USA) for 3 min at room temperature (RT).

### 4.4. Microscopy Analysis

Stained metaphase chromosomes and results of fluorescent in situ hybridization (FISH) were captured using a charge-coupled device (CCD)camera installed on an Axioplan 2 compound microscope (ZEISS, Germany) equipped with #49, #10, and #15 filter cubes (ZEISS, Germany) using AxioVision (ZEISS, Germany) or ISIS4 (METASystems GmbH, Germany) software at the Center for Microscopic Analysis of Biological Objects of SB RAS (Novosibirsk, Russia).

### 4.5. Morphometric Analysis

A range of morphological measurements (see Table 2) of metaphase chromosomes of *M. cliftonensis* and *M. mirumnovem* were carried out on captured images using MicroMeasure 3.3, as previously described [36].

### 4.6. Chromosome Microdissection and Microdissected DNA Probe Generation

The techniques for metaphase chromosome microdissection and DNA library generation using a whole-genome amplification kit were performed as described earlier [36,38,39]. Briefly, most microdissected DNA libraries were generated from one single copy of the whole chromosome, since this excludes possible mistakes in chromosome identification. For the generation of microdissected DNA libraries from whole individual chromosomes of *M. mirumnovem*, all chromosomes from a metaphase plate were isolated into individual tubes and treated according to standard protocol [36,38,39], using only metaphase plates with the most commonly observed 2n = 9 karyotype, avoiding metaphase plates with other karyotype variants (Figure 3b–l). After verification using FISH, the single-copy chromosome-specific DNA probes originating from homologous chromosomes (MMI2, MMI3, MMI4, and MMI5) were pooled and used for FISH experiments. In addition, two region-specific microdissected DNA probes were generated from 15 copies of the distal and proximal regions of the large paired metacentric chromosomes (MMI2) of *M. mirumnovem*, as was done previously for MLI1 of *M. lignano* [39]. We separately dissected and collected copies of the proximal and distal regions of both arms.

DNA labeling was performed according to a previously described technique [36,38,39] employing an additional 20 cycles of PCR using the Whole Genome Amplification 3 (WGA3) kit (Sigma-Aldrich, St. Louis, MO, USA) in the presence of Flu-12-dUTP, fluorescein-5(6)-carboxamidocaproyl-[5(3-aminoallyl)2′-deoxyuridine-5′-Triphosphate)] (Biosan, Novosibirsk, Russia) or TAMRA-5-dUTP, 5-tetramethylrhodamine-dUTP (Biosan). The DNA probes obtained earlier from chromosomes and chromosome regions of *M. lignano* [38,39] were also used for FISH experiments. All microdissected DNA probes used in this study are listed in Table 3.

### 4.7. Fluorescent In situ Hybridization

DNA probes for the detection of 5S and 28S rDNA clusters were prepared as previously described [38]. Briefly, to amplify a 1177 bp fragment of the 28S rDNA gene of *M. lignano*, WormA (5-GCGAATGGCTCATTAAATCAG-3) and WormB (5-CTTGTTACGACTTTTACTTCC-3) primers were used [40] and to amplify a 1645 bp fragment of the 5S rDNA gene of *M. lignano*, 5SF (5-CACCGGTTCTCGTCTGATCAC-3) and 5SR (3-CAACGTGGTATGGCCGTTG-5) primers were used [39]. The PCR product contained coding and non-transcribed (NTS) regions of the 5S rDNA. The 5S and 28S rDNA were labeled in additional PCR cycles [39]. The DNA probes specific to the clusters of rDNA of *M. lignano* were used on chromosomes of *M. lignano* and the other three *Macrostomum* species (*M. janickei*, *M. cliftonensis*, *M. mirumnovem*). We took into account the previously described method [36].

FISH experiments with microdissected DNA probes on metaphase chromosomes were carried out with partial suppression of repetitive DNA sequence hybridization. Due to the small body size of the studied species (body length ranging from 0.7–1.7 mm) [34], we could not obtain a sufficient amount of DNA for a Cot1/Cot2 DNA preparation (fraction of highly repetitive DNA sequences) in order to suppress the hybridization between labeled repetitive DNA in microdissected DNA probes and DNA repeats in metaphase chromosomes. Therefore, we modified the protocol for chromosomal in situ suppression hybridization (CISS) for partial suppression of repetitive DNA hybridization [38]. The single-copy chromosome-specific DNA probes derived from homologous chromosomes were pooled together after preliminary verification by FISH. This allowed an increase in the intensity of specific signals from FISH on corresponding chromosomes, while a low-intensity background signal on other chromosomes was also observed.

## Figures and Tables

**Table 1:** Single-worm karyotyping of specimens of the four studied *Macrostomum* species.

Line, Culture or Field Collection	Year Analyzed	*n*	Diploid Karyotype, *n* (%)	Aneuploid Variants of Karyotype, *n* (%)			References
(A) *M. lignano* (2*n* = 8, 2*n* = 9, 2*n* = 10, 2*n* = 11, and 2*n* = 12 karyotypes carry 2, 3, 4, 5, or 6 large metacentrics, respectively)							
Field collection			2*n* = 8	2*n* = 9	2*n* = 10	Other	
	2014	122	120 (98.4%)	1 (0.8%)	–	1 (0.8%)	[36]
DV1 (2003)			2*n* = 8	2*n* = 9	2*n* = 10	Other	
	2014	134	16 (11.9%)	77 (57.5%)	36 (26.9%)	5 (3.7%)	[36]
	2015	78	3 (3.8%)	23 (29.5%)	51 (65.4%)	1 (1.3%)	This study
DV1/10 (2015)			2*n* = 10	2*n* = 11	2*n* = 12	Other	
	2015	100	97 (97%)	1 (1%)	–	2 (2%)	[38]
	2016	100	96 (96%)	2(1%)	–	3 (3%)	[38]
	2017	94	89 (94.7%)	–	–	5 (5.3%)	This study
	2018	100	95 (95%)	5 (5%)	–	–	This study
(B) *M. janickei* (2*n* = 9, 2*n* = 10, and 2*n* = 11 karyotypes carry 3, 4, or 5 large metacentrics, respectively)							
Field collection			2*n* = 10	2*n* = 9	2*n* = 11	Other	
	2014	22	18 (81.8%)	2 (9.09%)	2 (9.09%)	–	[36]
2014 culture	2018	100	48 (48%)	–	39 (39%)	13 (13%)	This study
(C) *M. mirumnovem* (2*n* = 10 and 2*n* = 11 karyotypes carry 1 or 2 additional large metacentrics, respectively)							
2017 Culture			2*n* = 9	2*n* = 10	2*n* = 11	Other	
	2017	52	34 (65.4%)	1 (1.9%)	–	17 (32.7%)	This study
	2018	100	20 (20%)	26 (26%)	8 (8%)	46 (46%)	This study
(D) *M. cliftonensis* (no other karyotypes have so far been observed)							
2017 Culture			2*n* = 6	Other			
	2017	10	10 (100%)	–	–	–	This study
	2018	100	100 (100%)	-	-	-	This study

**Table 2:** Morphometric analysis of karyotypes of two newly analyzed *Macrostomum* species. (**A**) *M. mirumnovem* based on metaphase plates from worms with the 9,−MMI1 karyotype (*n* = 50); (**B**) *M. cliftonensis* based on its 2*n* = 6 metaphases (*n* = 50). Reported values represent mean ± 1 SD and include the absolute length (AL) and relative length (RL) of each chromosome, the length of the long arm (L) and short arm (S) and the arm ratio (R = L/S), and the centromeric index (CI = S/(L + S)). m, metacentric.

	AL (µm)	RL (%)	L (µm)	S (µm)	R	CI
(A) *Macrostomum mirumnovem*						
1	9.63 ± 0.46	40.00 ± 0.35	5.02 ± 0.44	4.53 ± 0.45	1.09 ± 0.67	0.48 ± 0.01 (m)
2	8.32 ± 0.59	34.87 ± 0.49	4.46 ± 0.47	3.93 ± 0.49	1.12 ± 0.07	0.46 ± 0.02 (m)
3	4.12 ± 0.45	16.80 ± 0.41	2.17 ± 0.25	1.94 ± 0.22	1.12 ± 0.09	0.47 ± 0.02 (m)
4	3.68 ± 0.39	15.03 ± 0.32	1.96 ± 0.23	1.72 ± 0.19	1.13 ± 0.09	0.47 ± 0.02 (m)
5	3.24 ± 0.38	13.29 ± 0.33	1.74 ± 0.24	1.49 ± 0.19	1.17 ± 0.15	0.46 ± 0.03 (m)
(B) *Macrostomum cliftonensis*						
1	4.27 ± 0.36	37.62 ± 0.57	2.3 ± 0.23	1.97 ± 0.16	1.18 ± 0.09	0.46 ± 0.02 (m)
2	3.71 ± 0.33	32.71 ± 0.48	1.93 ± 0.22	1.7 ± 0.21	1.16 ± 0.08	0.47 ± 0.02 (m)
3	3.31 ± 0.3	29.67 ± 0.38	1.76 ± 0.17	1.55 ± 0.16	1.15 ± 0.09	0.47 ± 0.02 (m)

**Table 3:** Microdissected region- and chromosome-specific DNA probes used in this study.

DNA Probe	Species	Original Chromosome or Chromosomal Region(s) *	*n* **	Generation of DNA Probe
*Mli2*	*M. lignano*	MLI2	15	[38]
*Mli3_4*	*M. lignano*	MLI3 and MLI4	15	[38]
*Mmi2med*	*M. mirumnovem*	Medial part of chromosome MMI2	15	This study
*Mmi2dist*	*M. mirumnovem*	Distal parts of p and q-arms of chromosome MMI2	15	This study
*Mmi1*	*M. mirumnovem*	MMI1	1	This study
*Mmi2*	*M. mirumnovem*	MMI2	2	This study
*Mmi3*	*M. mirumnovem*	MMI3	2	This study
*Mmi4*	*M. mirumnovem*	MMI4	2	This study
*Mmi5*	*M. mirumnovem*	MMI5	2	This study

* Chromosome names are designated with the first letter of the genus, two letters of the species (in capital letters), and the chromosome number in the karyotype (see Figure 1 for the chromosome numbers); ** collected copies of the chromosome or chromosome region(s).

## Supplementary Materials

Supplementary materials can be found at https://www.mdpi.com/1422-0067/21/2/680/s1.

## Abbreviations

WGD	Whole genome duplication
TE	Transposable element
