# Supplementary material for: Genome and Karyotype Reorganization after Whole Genome Duplication in Free-Living Flatworms of the Genus Macrostomum
Source: Int J Mol Sci. 2020 Jan 20;21(2):680. doi: 10.3390/ijms21020680 (PMC7013459; doi:10.3390/ijms21020680)
Supplement: Supplementary file 1 [file ijms-21-00680-s001.zip › Supplementary Material/Table S2.docx]

**Table S2:** Localization of specific fluorescent signals from microdissected DNA probes on different chromosomes of *M. mirumnovem*.

|  | **CHROMOSOMES** | | | | |
| --- | --- | --- | --- | --- | --- |
| ***DNA probe*** | **MMI1** | **MMI2** | **MMI3** | **MMI4** | **MMI5** |
| *Mmi2med* | weak fluorescence in medial part | medial part | pericentromeric region | pericentromeric region | pericentromeric region and regions on both sides of the region |
| *Mmi2dist* | weak fluorescence in distal parts of p- and q-arms | distal parts of p- and q-arms | dispersed fluorescent signals along chromosome | background signal | background signal |
| *Mmi1* | whole chromosome | pericentromeric region | pericentromeric region | pericentromeric region | pericentromeric region |
| *Mmi2* | dispersed signals | whole chromosome | pericentromeric region | background signal | background signal |
| *Mmi3* | signals from clusters in p-arm | signals from clusters in p-arm | whole chromosome | pericentromeric region | pericentromeric regions |
| *Mmi4* | p-arm | p-arm | background signal | whole chromosome | background signal |
| *Mmi5* | pericentromeric region; signals from clusters in p-arm | pericentromeric region | pericentromeric region | pericentromeric region | whole chromosome |
