# Supplementary material for: Genome and Karyotype Reorganization after Whole Genome Duplication in Free-Living Flatworms of the Genus Macrostomum
Source: Int J Mol Sci. 2020 Jan 20;21(2):680. doi: 10.3390/ijms21020680 (PMC7013459; doi:10.3390/ijms21020680)
Supplement: Supplementary file 1 [file ijms-21-00680-s001.zip › Supplementary Material/Table S3_final version.docx]

**Table S3:** The karyotype organization of some karyotype variants of *M. mirumnovem* based on FISH with microdissected DNA probes specific to chromosomes MMI1 and MMI2.

| **Chromosome number** | **Karyotype** | **No. of copies of large chromosomes** | | **No. of small chromosomes** | **B chromosomes** |
| --- | --- | --- | --- | --- | --- |
|  |  | **MMI1** | **MMI2** |  |  |
| 2n=8 | 8,-MMI1,-MMI2 | 1 | 1 | 6 |  |
| 2n=9 | 9,-MMI1 | 1 | 2 | 6 |  |
| 2n=10 | 10,-MMI1,+1B | 1 | 2 | 7 | 1 |
| 2n=10 | 10,-MMI1,-MMI2,+2B | 1 | 1 | 8 | 2 |
| 2n=11 | 11,-MMI1,+2B | 1 | 2 | 8 | 2 |
| 2n=11 | 11,+1B | 2 | 2 | 7 | 1 |
| 2n=12 | 12,+2B | 2 | 2 | 8 | 2 |
| 2n=12 | 12,+MMI2,+1B | 2 | 3 | 7 | 1 |
| 2n=13 | 13,+3B | 2 | 2 | 9 | 3 |
