# Supplementary material for: Genome and Karyotype Reorganization after Whole Genome Duplication in Free-Living Flatworms of the Genus Macrostomum
Source: Int J Mol Sci. 2020 Jan 20;21(2):680. doi: 10.3390/ijms21020680 (PMC7013459; doi:10.3390/ijms21020680)
Supplement: Supplementary file 1 [file ijms-21-00680-s001.zip › Supplementary Material/Table S1_final version.docx]

**Table S1**: List of observed karyotype variants revealed among the checked specimens of the *M. mirumnovem* culture.

| **Karyotype, 2n** | **Number of copies of large and small metacentric chromosomes** | | **Other chromosome morphotypes** | **N (%)** |
| --- | --- | --- | --- | --- |
|  | *large* | *small* |  |  |
| I. First karyotyping (2017) N=52 | | | | |
| 2n=7 | 1 | 6 | - | 1 (1.92%) |
| 2n=8 | 3 | 5 | - | 1 (1.92%) |
| 2n=9 | 3 | 6 | - | 34 (65.4%) |
| 2n=9+1B | 3 | 6 | one additional tiny chromosome | 7 (13.5%) |
| 2n=10 | 3 | 7 | - | 2 (3.85%) |
| 2n=10 | 4 | 6 | - | 1 (1.92%) |
| 2n=11 | 3 | 8 | - | 1 (1.92%) |
| 2n=12 | 3 | 9 | - | 3 (5.77%) |
| 2n=13 | 4 | 9 | - | 1 (1.92%) |
| 2n=14 | 6 | 8 | - | 1 (1.92%) |
| II. Karyotyping (2018) N=100 | | | | |
| 2n=5 | 2 | 3 | - | 2 (2%) |
| 2n=7 | 1 | 6 | - | 2 (2%) |
| 2n=8 | 1 | 7 | - | 1 (1%) |
| 2n=8 | 2 | 6 | - | 3 (3%) |
| 2n=8 | 3 | 5 | one medium-sized submetacentric | 1 (1%) |
| 2n=9 | 3 | 6 | - | 20 (20%) |
| 2n=9 | 4 | 5 | - | 1 (1%) |
| 2n=10 | 2 | 8 |  | 1 (1%) |
| 2n=10 | 4 | 6 | - | 26 (26%) |
| 2n=11 | 5 | 6 | - | 8 (8%) |
| 2n=12 | 3 | 9 | - | 1 (1%) |
| 2n=12 | 4 | 6 | two additional very small chromosomes | 1 (1)% |
| 2n=12 | 5 | 7 | - | 2 (2%) |
| 2n=13 | 3 | 10 | - | 1 (1%) |
| 2n=13 | 4 | 6 | three additional very small chromosomes | 2 (2%) |
| 2n=13 | 5 | 8 | - | 1 (1%) |
| 2n=13 | 6 | 7 | - | 1 (1%) |
| 2n=14 | 4 | 10 | - | 2 (2%) |
| 2n=14 | 5 | 9 | - | 2 (2%) |
| 2n=15 | 4 | 11 | - | 2 (2%) |
| 2n=15 | 5 | 10 | - | 2 (2%) |
| 2n=15 | 6 | 9 | - | 2 (2%) |
| 2n=16 | 4 | 12 | - | 1 (1%) |
| 2n=16 | 6 | 10 | - | 2 (2%) |
| 2n=16 | 8 | 8 | - | 1 (1%) |
| 2n=17 | 6 | 11 | - | 4 (4%) |
| 2n=17 | 5 | 12 | - | 2 (2%) |
| 2n=18 | 7 | 11 | - | 1 (1%) |
| 2n=19 | 6 | 13 | - | 2 (2%) |
| 2n=20 | 6 | 14 | - | 1 (1%) |
| 2n=21 | 6 | 15 | - | 1 (1%) |
| 2n=23 | 7 | 16 | - | 1 (1%) |
