# Supplementary figures and images for: Genome and Karyotype Reorganization after Whole Genome Duplication in Free-Living Flatworms of the Genus Macrostomum
Source: Int J Mol Sci. 2020 Jan 20;21(2):680. doi: 10.3390/ijms21020680 (PMC7013459; doi:10.3390/ijms21020680)

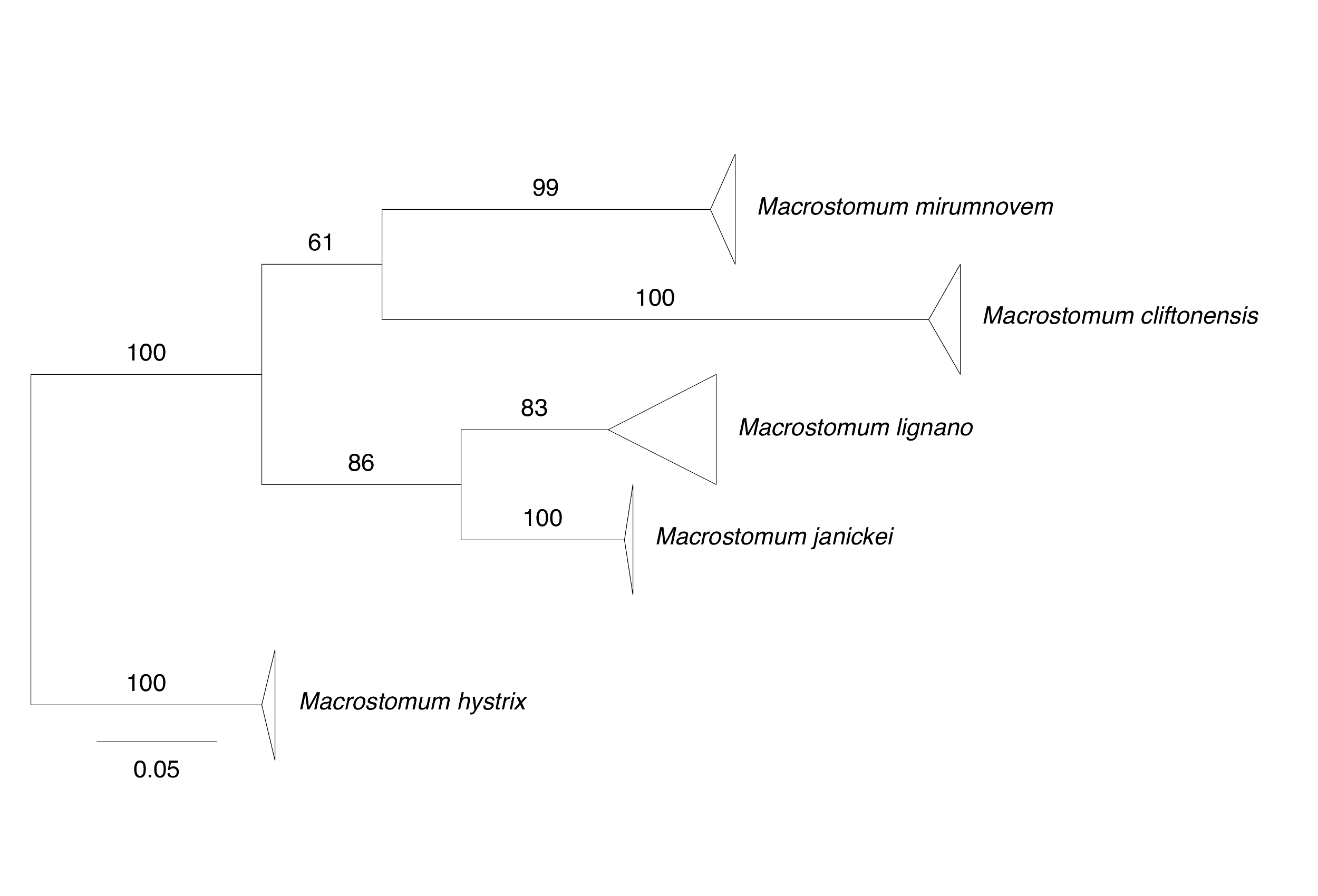

Supplement: Supplementary file 1 [file ijms-21-00680-s001.zip › Supplementary Material/Figure S1.tif]

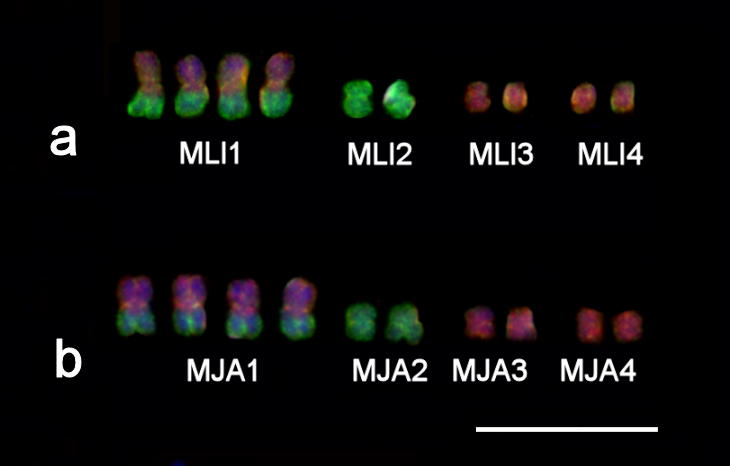

Supplement: Supplementary file 1 [file ijms-21-00680-s001.zip › Supplementary Material/Figure S2.tif]

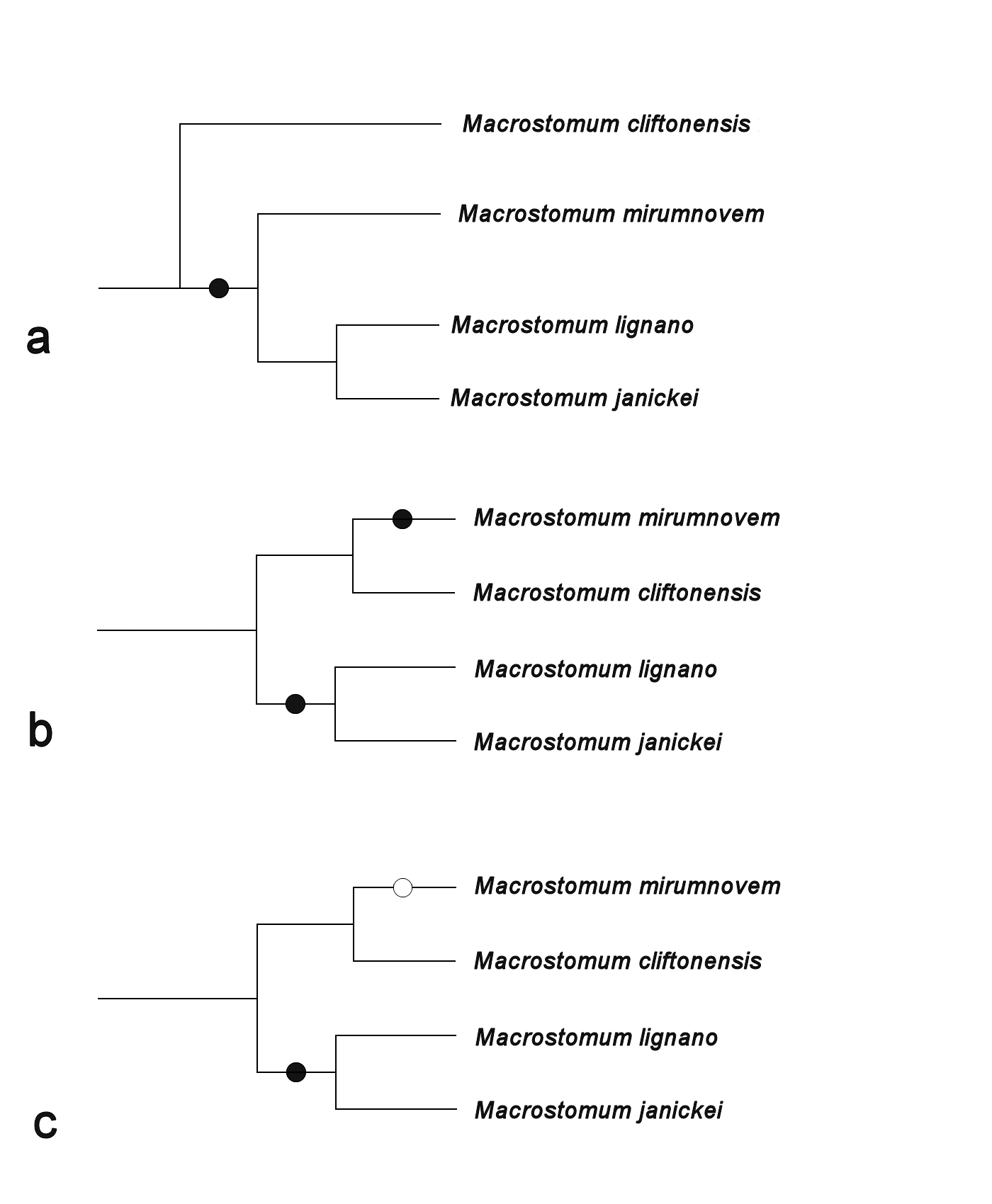

Supplement: Supplementary file 1 [file ijms-21-00680-s001.zip › Supplementary Material/Figure S3_final version.tif]
